# Application of zone classification in multiple intracranial aneurysmal subarachnoid hemorrhage treatment strategies

**DOI:** 10.1016/j.heliyon.2024.e26857

**Published:** 2024-02-22

**Authors:** Haonan Liu, Qian Xu, Hua Yang

**Affiliations:** a Department of Neurosurgery, The Affiliated Hospital of Guizhou Medical University, 28 Guiyi Street, Guiyang City, Guizhou Province, 550001, China; b Department of Medical Administration, Yancheng No.1 People's Hospital, 66 South Renmin Road, Yancheng City, Jiangsu Province, 224001, China

**Keywords:** Zone classification, Multiple intracranial aneurysm, Subarachnoid hemorrhage, Treatment

## Abstract

**Background:**

The options of surgical approach and treatment stage are two challenging treatment strategy issues with multiple intracranial aneurysmal subarachnoid hemorrhage (MIA-SAH).

**Methods:**

We retrospectively analyzed data from patients with MIA-SAH who underwent surgery in our center between January 1, 2014 and September 1, 2022. To define “zone classification”, the cranial cavity was divided into four zones by the planes of cerebral falx and tentorium cerebelli. Aneurysms isolated to one zone were defined as zone classification I; those crossing two zones were defined as zone classification II; those crossing three zones were defined as zone classification III; and those crossing four zones were defined as zone classification IV. General and aneurysmal-related characteristics of patients with different zone classifications were collected and compared between two surgical approaches. Multivariate logistic regression analysis was used to identify factors independently associated with multistage treatment options.

**Results:**

A total of 226 patients with 523 aneurysms were included. The proportion of patients undergoing endovascular treatment increased with higher zone classification (I: 85.4%; II: 94.0%; III: 100.0%; IV: 100.0%). The proportion of patients receiving one-stage treatment decreased with higher zone classification (I: 60.2%; II: 33.6%; III: 0.0%; IV: 0.0%). Compared with patients undergoing microsurgical clipping, more patients undergoing endovascular treatment had zone classification II–IV (56.9% *vs.* 31.8%, *p* = 0.025). Zone classification II–IV (odds ratio [OR] = 3.821, 95% confidence interval [CI]: 2.041–7.154, *p* < 0.001), endovascular treatment (OR = 8.756, 95% CI: 2.589–29.609, *p* < 0.001), and size of all unruptured aneurysms <3 mm (OR = 4.531, 95% CI: 2.315–8.871, *p* < 0.001) were each independently associated with multistage treatment.

**Conclusions:**

Zone classification provides a new idea in MIA-SAH treatment strategies, especially regarding surgical approach and treatment stage options.

## 1 Introduction

Subarachnoid hemorrhage (SAH), a very dangerous stroke subtype, is usually caused by rupture of intracranial aneurysms (IAs) and currently has unsatisfactory prognosis [1]. Surgery, including microsurgical clipping and endovascular treatment, is the preferred treatment for patients with aneurysmal subarachnoid hemorrhage (aSAH) [2]. The one-third of patients with aSAH also have multiple intracranial aneurysms (MIAs), with higher short-term morbidity compared with patients with a single IA [3]. There may be demographic and clinical differences between patients with SAH presenting with single or multiple aneurysms [4,5]. Additive effects of coexisting aneurysms may also increase SAH risk in patients with MIAs [6]. The options of surgical approach and treatment stage are two challenging treatment strategy issues with multiple intracranial aneurysmal subarachnoid hemorrhage (MIA-SAH).

In 2001, we speculated about the use of MIA zone classification, based on our report of 20 patients [7]. Briefly, patients with MIAs were classified by IA locations. Unfortunately, since this report was published in a Chinese language journal and did not include detailed criteria for zone classifications, this issue was not investigated further during the subsequent 22 years. Thus, our aim herein was to investigate the application of zone classification in MIA-SAH treatment strategies.

## 2 Methods

### 2.1 Patients

We retrospectively analyzed data from patients with MIA-SAH who underwent surgery in our center between January 1, 2014 and September 1, 2022. All procedures were performed in accordance with The Code of Ethics of the World Medical Association (Declaration of Helsinki). The study was approved by the Ethics Committee of Guizhou Medical University with a waiver of informed consent (No. 2022 [291]). The inclusion criteria were: (1) spontaneous SAH observed on head computed tomography or lumbar puncture at admission; (2) MIAs confirmed by digital subtraction angiography or surgery; (3) age >18 years; and (4) undergoing microsurgical clipping or endovascular treatment. The exclusion criteria were: (1) SAH with other causes; (2) a history of stroke or other diseases that may bias study results; and (3) incomplete data.

### 2.2 Data collection

All data were obtained from electronic medical records: age, sex, body mass index, systolic and diastolic blood pressure at admission, Hunt and Hess grade at admission, modified Fisher grade, surgical approaches (microsurgical clipping and endovascular treatment, defined as the final treatment option for the ruptured aneurysm), all aneurysm features (location, number, size, shape, and treatment), treatment stage (one-stage treatment for all aneurysms and multistage treatment), leukocyte count and hemoglobin on admission, complications (delayed cerebral ischemia, rebleeding, and hydrocephalus), and Glasgow Outcome Scale [8] score three months after surgery (score of 1–3, adverse outcomes). Flow diversion was also classified as endovascular treatment. For complex cases, surgery was performed in the hybrid operating room.

### 2.3 Zone classification

To define “zone classification”, the cranial cavity was divided into four zones by the planes of cerebral falx and tentorium cerebelli [7]. IAs isolated to one zone were defined as zone classification I; those crossing two zones were defined as zone classification II; those crossing three zones were defined as zone classification III; and those crossing four zones were defined as zone classification IV.

The anterior communicating artery was divided, following tradition, into three parts: left, middle, and right. Cases with aneurysms located in the (middle) anterior communicating artery and basilar artery were defined as the higher classification option. For example, a patient with *1 basilar artery aneurysm* + *1 right vertebral artery aneurysm* was categorized as zone classification II. In addition, if a patient had one aneurysm located at A3 segment or more of the anterior cerebral artery and another at M3 segment or more of the middle cerebral artery on the same side, they were categorized as zone classification II.

### 2.4 Statistical analysis

Categorical variables are expressed as frequencies and percentages and were compared, as appropriate, by chi-square or Fisher's exact probability test. Normally distributed continuous variables are expressed as means ± standard deviations and were compared using independent-samples *t*-tests. Non-normally distributed continuous variables are presented as medians and interquartile ranges (25th, 75th) and were compared using Mann–Whitney *U*-tests. Appropriate variables were selected for logistic regression analysis, based on statistical significance. Univariate logistic regression was performed first, and variables with *p* < 0.05 were then included in the multivariate logistic regression analysis (using enter method) to identify factors independently associated with multistage treatment options. *p* < 0.05 was considered statistically significant. All statistical analyses were performed using SPSS version 23.0 (IBM Corporation, Armonk, NY, USA).

## 3 Results

A total of 226 patients were included in analyses. Their mean age was 55 years, and 149 were female (65.9%). These patients had a total of 523 aneurysms, among which the top five locations were: internal carotid artery (133 aneurysms, 25.4%); middle cerebral artery (103, 19.7%); posterior communicating artery (100, 19.1%); anterior communicating artery (87, 16.6%); and anterior cerebral artery (24, 4.6%). There were 152 aneurysms of <3 mm, 213 of 3–4.9 mm, 132 of 5–9.9 mm, 21 of 10–14.9 mm, 4 of 15–19.9 mm, 1 of 20–24.9 mm, and none ≥25 mm. One-hundred-seventy-eight patients (78.8%) had two aneurysms, among whom 42 had mirror aneurysms. General and aneurysmal-related characteristics of patients with different zone classifications were shown in Table 1.

**Table 1:** General and aneurysmal-related characteristics of patients with different zone classifications.

	I (*n* = 103)	II (*n* = 116)	III (*n* = 6)	IV (*n* = 1)
Age >60 years, *n* (%)	29 (28.2)	38 (32.8)	1 (16.7)	0 (0.0)
Female, *n* (%)	60 (58.3)	85 (73.3)	3 (50.0)	1 (100.0)
Hunt–Hess grade, *n* (%)				
1	10 (9.7)	12 (10.3)	0 (0.0)	1 (100.0)
2	48 (46.6)	61 (52.6)	2 (33.3)	0 (0.0)
3	29 (28.2)	31 (26.7)	2 (33.3)	0 (0.0)
4	15 (14.6)	10 (8.6)	1 (16.7)	0 (0.0)
5	1 (1.0)	2 (1.7)	1 (16.7)	0 (0.0)
Modified Fisher grade, *n* (%)				
0	1 (1.0)	4 (3.4)	0 (0.0)	0 (0.0)
1	5 (4.9)	6 (5.2)	0 (0.0)	0 (0.0)
2	65 (63.1)	72 (62.1)	3 (50.0)	1 (100.0)
3	14 (13.6)	17 (14.7)	1 (16.7)	0 (0.0)
4	18 (17.5)	17 (14.7)	2 (33.3)	0 (0.0)
Number of aneurysms, *n* (%)				
2	86 (83.5)	92 (79.3)	–	–
3	13 (12.6)	17 (14.7)	2 (33.3)	–
4	4 (3.9)	5 (4.3)	1 (16.7)	1 (100.0)
5	0 (0.0)	1 (0.9)	2 (33.3)	0 (0.0)
6	0 (0.0)	1 (0.9)	1 (16.7)	0 (0.0)
Ruptured aneurysm located in the posterior circulation, *n* (%)	11 (10.7)	12 (10.3)	0 (0.0)	0 (0.0)
Surgical approach for the ruptured aneurysm, *n* (%)				
Microsurgical clipping	15 (14.6)	7 (6.0)	0 (0.0)	0 (0.0)
Endovascular treatment	88 (85.4)	109 (94.0)	6 (100.0)	1 (100.0)
One-stage treatment, *n* (%)	62 (60.2)	39 (33.6)	0 (0.0)	0 (0.0)
GOS score after three months, *n* (%)				
1	8 (7.8)	10 (8.6)	1 (16.7)	0 (0.0)
2	16 (15.5)	19 (16.4)	0 (0.0)	0 (0.0)
3	19 (18.4)	12 (10.3)	2 (33.3)	0 (0.0)
4	18 (17.5)	28 (24.1)	2 (33.3)	0 (0.0)
5	42 (40.8)	47 (40.5)	1 (16.7)	1 (100.0)

GOS: Glasgow Outcome Scale.

In total, 227 aneurysms were found in 103 patients with zone classification I, 266 in 116 patients with zone classification II, 26 in 6 patients with zone classification III, and 4 in 1 patient with zone classification IV. The aneurysms’ common locations within zone classifications were shown in Supplementary Table 1.

Twenty-two patients (9.7%) underwent microsurgical clipping, while the others underwent endovascular treatment. The proportion of patients undergoing endovascular treatment increased with higher zone classification (I: 85.4%; II: 94.0%; III: 100.0%; IV: 100.0%). One-hundred-one patients (44.7%) received one-stage treatment for all aneurysms, while the others received multistage treatment. The proportion of patients receiving one-stage treatment for all aneurysms decreased with higher zone classification (I: 60.2%; II: 33.6%; III: 0.0%; IV: 0.0%).

Comparisons of clinical characteristics and outcomes of patients who underwent microsurgical clipping or endovascular treatment were shown in Table 2. Compared with patients undergoing microsurgical clipping, patients undergoing endovascular treatment had more zone classification II–IV (56.9% *vs.* 31.8%, *p* = 0.025), larger ruptured aneurysm sizes (5.0 mm *vs.* 3.9 mm, *p* = 0.030), and less rebleeding (6.9% *vs.* 22.7%, *p* = 0.032). There was not a significant difference between patients undergoing microsurgical clipping or endovascular treatment with respect to adverse outcomes after three months (*p* = 0.243).

**Table 2:** Comparison of clinical characteristics and outcomes in patients undergoing microsurgical clipping and endovascular treatment.

	Microsurgical clipping (*n* = 22)	Endovascular treatment (*n* = 204)	*p*-value
Systolic blood pressure at admission, mmHg	146.0 (132.8, 153.0)	149.0 (134.0, 169.0)	0.217
Diastolic blood pressure at admission, mmHg	84.9 ± 13.7	89.4 ± 14.0	0.151
Hunt–Hess grade, *n* (%)			0.701
1–3	18 (81.8)	178 (87.3)	
4–5	4 (18.2)	26 (12.7)	
Modified Fisher grade, *n* (%)			0.110
0–2	12 (54.5)	145 (71.1)	
3–4	10 (45.5)	59 (28.9)	
Intraventricular hemorrhage, *n* (%)	11 (50.0)	99 (48.5)	0.896
Intracerebral hematoma, *n* (%)	7 (31.8)	36 (17.6)	0.186
Ruptured aneurysm located in the posterior circulation, *n* (%)	0 (0.0)	23 (11.3)	0.197
Zone classification II–IV, *n* (%)	7 (31.8)	116 (56.9)	0.025*
Size of the ruptured aneurysm, mm	3.9 (3.0, 5.2)	5.0 (3.7, 6.7)	0.030*
Irregular shape of the ruptured aneurysm, *n* (%)	9 (40.9)	59 (28.9)	0.244
Delayed cerebral ischemia, *n* (%)	5 (22.7)	75 (36.8)	0.191
Rebleeding, *n* (%)	5 (22.7)	14 (6.9)	0.032*
Hydrocephalus, *n* (%)	4 (18.2)	32 (15.7)	1.000
Adverse outcomes after three months, *n* (%)	11 (50.0)	76 (37.3)	0.243

*p < 0.05.

Comparisons of general features, clinical characteristics, and outcomes of patients who received one-stage or multistage treatment were shown in Table 3. Compared with patients who received one-stage treatment, patients who received multistage treatment were less likely to have undergone microsurgical clipping (3.2% *vs.* 17.8%, *p* < 0.001), had more zone classification II–IV (67.2% *vs.* 38.6%, *p* < 0.001), a higher rate of unruptured aneurysms of <3 mm (51.2% *vs.* 28.7%, *p* < 0.001), and a higher rate of regular-shape unruptured aneurysms (77.6% *vs.* 64.4%, *p* = 0.028). There was not a significant between-group difference in complications or adverse outcomes after three months (*p* = 0.159).

**Table 3:** Comparison of general features, clinical characteristics, and outcomes in patients receiving one-stage or multi-stage treatment.

	Total (*n* = 226)	One-stage treatment (*n* = 101)	Multi-stage treatment (*n* = 125)	*p*-value
Age >60 years, *n* (%)	68 (30.1)	25 (24.8)	43 (34.4)	0.116
Female, *n* (%)	149 (65.9)	62 (61.4)	87 (69.6)	0.195
Body mass index	22.9 (20.9, 25.5)	22.3 (20.8, 25.0)	23.1 (21.0, 25.9)	0.403
Systolic blood pressure at admission, mmHg	150.6 ± 25.8	150.2 ± 27.7	150.9 ± 24.3	0.824
Diastolic blood pressure at admission, mmHg	88.9 ± 14.0	88.6 ± 14.0	89.2 ± 14.0	0.784
Hunt–Hess grade 4–5, *n* (%)	30 (13.3)	17 (16.8)	13 (10.4)	0.157
Modified Fisher grade 3–4, *n* (%)	69 (30.5)	31 (30.7)	38 (30.4)	0.962
Microsurgical clipping, *n* (%)	22 (9.7)	18 (17.8)	4 (3.2)	<0.001*
Ruptured aneurysm located in the posterior circulation, *n* (%)	23 (10.2)	11 (10.9)	12 (9.6)	0.750
Zone classification II–IV, *n* (%)	123 (54.4)	39 (38.6)	84 (67.2)	<0.001*
Size of the ruptured aneurysm, mm	4.8 (3.6, 6.4)	4.5 (3.6, 7.0)	4.9 (3.6, 6.2)	0.684
Irregular shape of the ruptured aneurysm, *n* (%)	68 (30.1)	32 (31.7)	36 (28.8)	0.638
Size of all unruptured aneurysms <3 mm, *n* (%)	93 (41.2)	29 (28.7)	64 (51.2)	<0.001*
Shape of all unruptured aneurysms were regular, *n* (%)	162 (71.7)	65 (64.4)	97 (77.6)	0.028*
Leukocyte count, × 10^9^/L	11.0 (8.8, 13.8)	10.8 (8.6, 13.3)	11.3 (8.8, 14.2)	0.382
Hemoglobin, g/L	134.0 (124.0, 146.0)	135.0 (122.0, 146.0)	134.0 (124.0, 145.0)	0.760
Delayed cerebral ischemia, *n* (%)	80 (35.4)	39 (38.6)	41 (32.8)	0.364
Rebleeding, *n* (%)	19 (8.4)	8 (7.9)	11 (8.8)	0.813
Hydrocephalus, *n* (%)	36 (15.9)	16 (15.8)	20 (16.0)	0.974
Adverse outcomes after three months, *n* (%)	87 (38.5)	44 (43.6)	43 (34.4)	0.159

*p < 0.05.

Multivariate logistic regression analysis revealed that zone classification II–IV (odds ratio [OR] = 3.821, 95% confidence interval [CI]: 2.041–7.154, *p* < 0.001), endovascular treatment (OR = 8.756, 95% CI: 2.589–29.609, *p* < 0.001), and size of all unruptured aneurysms <3 mm (OR = 4.531, 95% CI: 2.315–8.871, *p* < 0.001) were each independently associated with multistage treatment options, as shown in Table 4.

**Table 4:** Factors independently associated with multi-stage treatment options.

Variable	Univariate regression		Multivariate regression	
	OR (95% CI)	*P*-value	OR (95% CI)	*p*-value
Zone classification II–IV	3.257 (1.884–5.632)	<0.001*	3.821 (2.041–7.154)	<0.001*
Endovascular treatment	6.560 (2.143–20.083)	<0.001*	8.756 (2.589–29.609)	<0.001*
Size of all unruptured aneurysms <3 mm	2.605 (1.494–4.541)	<0.001*	4.531 (2.315–8.871)	<0.001*
Shape of all unruptured aneurysms were regular	1.919 (1.069–3.445)	0.029*	1.279 (0.666–2.457)	0.459

CI: confidence interval; OR: odds ratio. *p < 0.05.

## 4 Discussion

To our knowledge, this was the first study to apply zone classification to MIA-SAH surgical approach and treatment stage. Herein, the zone classification operational definition and special cases were detailed. The study's main findings were: (1) Patients undergoing endovascular treatment had more zone classification II–IV than those undergoing microsurgical clipping; and (2) Zone classification II–IV, endovascular treatment, and sizes of all unruptured aneurysms <3 mm were independently associated with multistage treatment. Based on these findings, we recommend using zone classification in MIA-SAH treatment strategies.

MIA-SAH treatment is highly challenging, and the standard surgical treatment remains controversial. A recent meta-analysis [9] revealed that female sex, older age, arterial hypertension, smoking, and familial intracranial aneurysm are major MIA risk factors, but that factors related to risk of rupture remain controversial. Roethlisberger et al. [10] found that the location of the ruptured index aneurysm was correlated with the probability of finding bystander aneurysms and predicted their likely anatomic distribution. Surgical approach and treatment stage options are two especially difficult issues in strategizing MIA-SAH treatment. Unruptured intracranial aneurysms (UIAs) in patients with MIA-SAH may differ from those with a single UIA because the former may be more unstable.

Most patients at our center underwent endovascular treatment. A retrospective study revealed that endovascular embolization may be a better approach to MIAs than surgical clipping [11]. The proportion of patients undergoing endovascular treatment increased with higher zone classification, suggesting that the more scattered the aneurysm distribution, the more suitable endovascular treatment becomes. However, microsurgical clipping retains classic and unique advantages. Combined microsurgical and endovascular management of complex intracranial aneurysms has been applied at many hospitals, including our center [12], because MIAs require tailored, individual approaches to aneurysmal occlusion [13].

One-stage treatment for all aneurysms is ideal, as it can greatly reduce the chance of future ruptures of untreated aneurysms. However, when one-stage treatment for all aneurysms involves technical difficulties and complications, multistage treatment may be safer. Herein, with increased zone classification, technical difficulties and potential complications also increased; thus, the proportion of patients receiving one-stage treatment for all aneurysms decreased. Seo et al. [14] found that one-stage multiple craniotomies for MIAs was safe and economical. Hong et al. [15] also demonstrated that one-stage clipping of MIAs showed satisfactory treatment outcomes. Andic et al. [16] believed that one-stage MIA treatment with combined endovascular techniques was technically feasible but that its safety due to relatively high complication rates remained controversial (i.e., because unruptured tiny aneurysms weighted the low annual spontaneous rupture rate and potential surgical risks) [17]. A long-term follow-up study [18] revealed that patients with moderate or severe disability after SAH have a relatively low risk of UIA rupture. Furthermore, over shorter follow-up, intervention and observation appear similarly safe for bystander aneurysms in MIAs [19].

In our center, determining which aneurysm is at risk of ruptured depends mainly on clinical experience, with SAH location and morphology serving as the two most important factors. While assessing risk for aneurysm rupture is highly complex, researchers have addressed this issue [20,21]. Some scoring systems for patients with MIAs have proven weak [[22], [23], [24]]. Neyazi and colleagues [25] concluded that many established morphological and hemodynamical parameters have little predictive value for aneurysm rupture in patients with MIAs. Shojima et al. [26] asserted that the largest among coexisting aneurysms was most likely to rupture. UIA size may be another factor for predicting rupture [27] and irregular shape might identify aneurysms that will rupture better than size in patients with MIA-SAH [28,29]. However, Orning et al. [30] stated that aneurysm morphology may not, in many cases, be used to reliably determine rupture site. This cumulative evidence indicates that much work remains toward identifying aneurysms likely to rupture.

This study was not without limitations. First, it was a single-center study, so the results need to be verified in other centers. Second, it was retrospective and thus the aneurysm treatments were not controlled. Finally, while its sample size was 226, only 22 patients underwent microsurgical clipping (probably indicating a selection bias), making it difficult to carry out multivariate logistic regression analysis of surgical approaches.

## 5 Conclusions

Zone classification provides a new idea in MIA-SAH treatment strategies, especially regarding surgical approach and treatment stage options. The proportion of patients undergoing endovascular treatment increased, and those receiving one-stage treatment for all aneurysms decreased, at higher zone classifications. Patients undergoing endovascular treatment had more zone classification II–IV than those undergoing microsurgical clipping. Zone classification II–IV, endovascular treatment, and sizes of all unruptured aneurysms <3 mm were independently associated with multistage treatment options. Based on this evidence, we recommend using zone classification in developing MIA-SAH treatment strategies.

## Funding

This work was supported by the “Science and Technology Planning Project of Guizhou Province” ([2016] 2905), “Key Discipline Construction Project of Guizhou Province” (2011 (52) - 02), and “Guizhou Provincial Science and Technology Innovation Talent Team Project” ((2020) 5014).

## Data availability

The datasets used and analyzed in the current study are available from the corresponding author on reasonable request.

## Ethical approval

The Ethics Committee of Guizhou Medical University approved this study and waived the requirement for informed consent because this was a retrospective study and the data were deidentified [approval number: 2022 (291)].

## Informed consent

For this type of study, formal consent is not required.

## CRediT authorship contribution statement

**Haonan Liu:** Data curation, Formal analysis, Investigation, Methodology, Resources, Software, Supervision, Validation, Visualization, Writing – original draft. **Qian Xu:** Data curation, Formal analysis, Investigation, Methodology, Resources, Software, Supervision, Validation, Visualization, Writing – review & editing. **Hua Yang:** Conceptualization, Funding acquisition, Project administration, Resources, Writing – review & editing.

## Declaration of competing interest

The authors declare that they have no known competing financial interests or personal relationships that could have appeared to influence the work reported in this paper.
