# Supplementary material for: Application of zone classification in multiple intracranial aneurysmal subarachnoid hemorrhage treatment strategies
Source: Heliyon. 2024 Feb 22;10(5):e26857. doi: 10.1016/j.heliyon.2024.e26857 (PMC10904235; doi:10.1016/j.heliyon.2024.e26857)
Supplement: Multimedia component 1 [file mmc1.doc]

**Supplementary Table 1.** Aneurysms’ common locations within zone classifications.

| Zone Classifications | I | II | III | IV |
| --- | --- | --- | --- | --- |
| Most Common | 2 left ACoA aneurysms | 1 left PCoA aneurysm + 1 right PCoA aneurysm | 1 left ICA aneurysm + 1 right ICA aneurysm + 1 BA aneurysm | 1 left ICA aneurysm + 1 right ICA aneurysm + 1 left VA aneurysm + 1 right VA aneurysm |
| Second Common | 1 left ACoA aneurysm + 1 left MCA aneurysm | 1 left ICA aneurysm + 1 right ICA aneurysm | 1 left MCA aneurysm + 1 right MCA aneurysm + 1 BA aneurysm | - |
| Third Common | 1 right ACoA aneurysm + 1 right ICA aneurysm | 1 left MCA aneurysm + 1 right MCA aneurysm | 1 left MCA aneurysm + 1 right MCA aneurysm + 1 left PCoA aneurysm + 1 BA aneurysm | - |

ACoA: anterior communicating artery; PCoA: posterior communicating artery; MCA: middle cerebral artery; ICA: internal carotid artery; BA: basilar artery; VA: vertebral artery.
